# An In Vitro Alveolar Model Allows for the Rapid Assessment of Particles for Respiratory Sensitization Potential

**DOI:** 10.3390/ijms241210104

**Published:** 2023-06-14

**Authors:** Matthew Gibb, Christie M. Sayes

**Affiliations:** 1 Institute of Biomedical Studies, Baylor University, Waco, TX 76798, USA; matthew_gibb@baylor.edu; 2 Department of Environmental Science, Baylor University, Waco, TX 76798, USA

**Keywords:** sensitization, pulmonary exposure, immunotoxicology, in vitro, dendritic cells, cellular activation

## Abstract

Dust, both industrial and household, contains particulates that can reach the most distal aspects of the lung. Silica and nickel compounds are two such particulates and have known profiles of poor health outcomes. While silica is well-characterized, nickel compounds still need to be fully understood for their potential to cause long-term immune responses in the lungs. To assess these hazards and decrease animal numbers used in testing, investigations that lead to verifiable in vitro methods are needed. To understand the implications of these two compounds reaching the distal aspect of the lungs, the alveoli, an architecturally relevant alveolar model consisting of epithelial cells, macrophages, and dendritic cells in a maintained submerged system, was utilized for high throughput testing. Exposures include crystalline silica (SiO_2_) and nickel oxide (NiO). The endpoints measured included mitochondrial reactive oxygen species and cytostructural changes assessed via confocal laser scanning microscopy; cell morphology evaluated via scanning electron microscopy; biochemical reactions assessed via protein arrays; transcriptome assessed via gene arrays, and cell surface activation markers evaluated via flow cytometry. The results showed that, compared to untreated cultures, NiO increased markers for dendritic cell activation, trafficking, and antigen presentation; oxidative stress and cytoskeletal changes, and gene and cytokine expression of neutrophil and other leukocyte chemoattractants. The chemokines and cytokines CCL3, CCL7, CXCL5, IL-6, and IL-8 were identified as potential biomarkers of respiratory sensitization.

## 1. Introduction

The lungs are a complex network of cell types involving cellular crosstalk, communication, and varying motions (e.g., mucociliary ladder and surfactants). Because of this heterogeneity of cellular structure, one of the most critical aspects of pulmonary in vitro study is the ability to adequately maintain relevant cellular architecture in selected models [1].

With the primary function of the lungs being gas exchange, it is critically important to test and assess the potential for poor health outcomes associated with inhaled air. Inhaled air can consist of chemicals and particulates that, depending on various physicochemical properties, can deposit on cells and affect cellular responses throughout the respiratory system [2]. Of the known potential health outcomes, allergic-type reactions are of primary concern, as they can lead to life-long issues or be severe enough to cause anaphylaxis and possibly death. Respiratory sensitization refers to the onset of inflammatory responses, including airway hypersensitivity, asthma, bronchiolitis, and more [3].

Sensitization, whether in the skin or lungs, involves two consecutive steps: (i) Induction, where an exposure leads to a cascade of innate and adaptive cells activating and maturating to provide a specific elevated immune response on secondary exposure; and (ii) Elicitation, where an exacerbated immune response occurs on second exposure, leading to a variety of inflammation, as seen in acute and chronic asthma, as well as anaphylaxis [4]. Typically, the order of events requires a minimum of two exposures for any allergic reaction to occur [5].

Currently, most research into respiratory sensitization has focused on low molecular weight (LMW) and high molecular weight (HMW) chemicals [6]. Most known sensitizers, including LMW chemicals, are too small to create an immune response independently and require protein binding to elicit an immune response. The sensitizer, a hapten, and a protein bind are needed to form a hapten–protein complex recognizable by the immune system [5,7]. Importantly, alveolar macrophages and surrounding epithelial cells can provide the proteins necessary to form these complexes [8].

Identifying and understanding the mechanisms associated with respiratory sensitization has primarily focused on rodent studies or gathered from population-level studies in humans [9,10,11]. These kinds of studies are costly, time-consuming, and need more translatability to humans [9,10,11,12,13]. The ability to utilize human cells in vitro has helped to recapitulate human responses better. Furthermore, the ability to better mimic in vivo architecture while working with human-derived cells allows for increased capacity for direct translation from in vitro to in vivo outcomes without dealing with the dynamic nature of in vivo studies [1,14,15].

Crystalline silica (SiO_2_) is ubiquitous in the earth’s crust and is known to lead to adverse pulmonary health through silicosis, where trapped silica lead to inflammation; scarring; lung cancer; chronic obstructive pulmonary disease (COPD), and kidney disease [16]. Common exposures include industries that involve sand, mortar, stone, and concrete, where the respirable form of SiO_2_ is created from sawing, drilling, crushing, grinding, and cutting [17]. SiO_2_ is not known to lead to respiratory sensitization despite decades of research on human populations after exposure; however, it is known to be a respiratory irritant that leads to oxidative stress for all cell types within the lungs on exposure [18].

Nanomaterials have been shown to target immune cells to varying degrees, and with nanometals being produced in vast quantities, understanding their effect on human health is imperative [19,20,21,22,23]. Nickel compounds, specifically nickel oxide (NiO), have been shown to induce adverse respiratory effects such as asthma and eosinophilic inflammation [24]. IgE antibody tests are frequently utilized to assess for a portion of sensitizing reactions. NiO has been shown to increase serum IgE levels when using bulk and nano-scale material [24]. Animal modeling can provide insights into possible human responses, but difficulties arise when investigating the respiratory sensitizing potential. For instance, rats require a much higher level of the test compound to elicit a broncho-restrictive response, and guinea pigs will produce IgG1 rather than IgE to known respiratory allergens [6,25,26].

Sensitization can occur anywhere within the lungs; however, a single model is currently incapable of recapitulating the lungs due to the complexity of the lung cellular architecture. Because gas exchange occurs at the alveolar space, understanding immune responses in this compartment is crucial to potential preventatives, interventions, and treatments. Within the alveolar region, there are three main cell types: epithelial cells (both Type I and II) and immune cells, specifically alveolar macrophages (AMs) in the luminal space and dendritic cells (DCs), which are scattered among the basement membrane [27].

Previously, an easy, reliable, and verified cell culture model that can be adopted by any lab capable of performing molecular toxicology studies was used to study a known chemical respiratory sensitizer, isophorone diisocyanate (IPDI), and a known cell activator, phorbal 12-myristate 13-acetate (PMA) and ionomycin [6]. Here, the same model and endpoints (morphology, biochemical perturbations, and transcriptome) were chosen to assess if the model can differentiate between a known irritating respiratory particulate (SiO_2_) and a suspected sensitizing respiratory particulate (NiO). Like the previous study, the results suggest that multiple techniques and endpoints can show objective distinctions in immune responses after different particulate exposures.

## 2. Results

**Figure 1:**
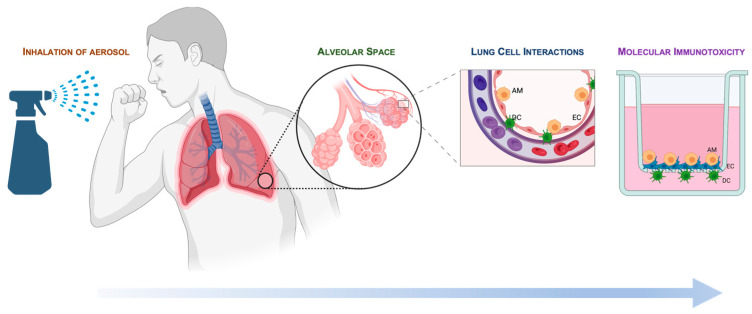
Model development. The model is based on real-world exposure to aerosol, where the final deposition is in the alveolar space. The model depicts in vivo architecture. Cultured cells are arranged in a Transwell^®^ and include differentiated U937 cells (as alveolar macrophages, AMs), A549 cells (as type 1 epithelial cells, ECs), and JawsII cells (as dendritic cells, DCs).

The model setup is based on in vivo alveolar cellular architecture, which contains epithelial cells (ECs), alveolar macrophages (AMs), and dendritic cells (DCs). Figure 1 shows the developmental process from aerosol exposure to in vitro recapitulation of cellular components and location in the Transwell^®^. The characteristics of toxicology and immunology assessments, as well as basal properties, have been studied. This includes evaluating transepithelial electrical resistance (TEER) [28,29,30,31,32].

**Figure 2:**
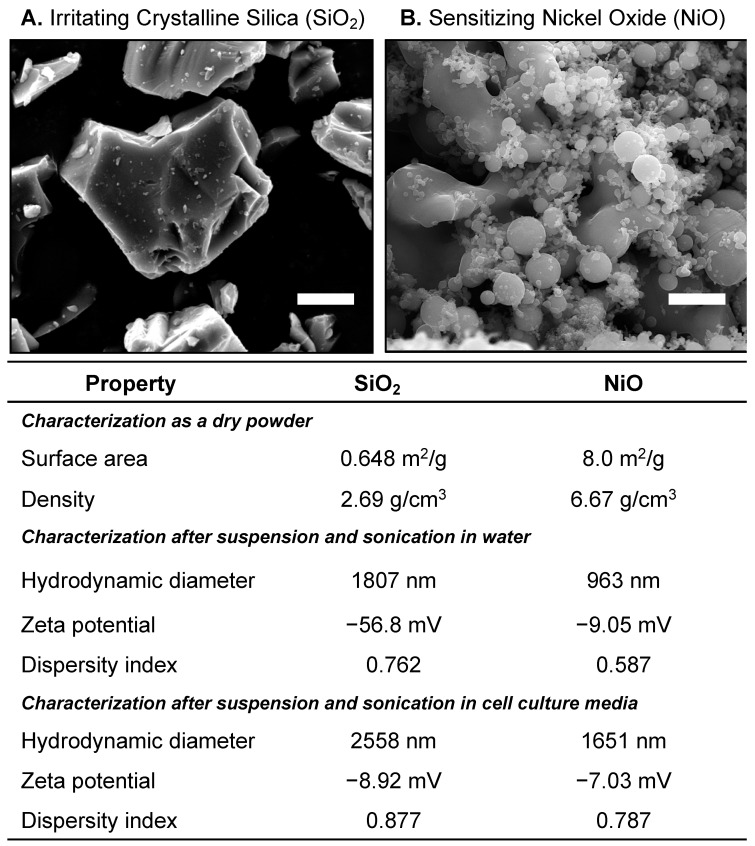
Physicochemical characterization of the materials used in the study. (**A**) Scanning electron microscopy (SEM) image of irritating crystalline silica (SiO_2_), (**B**) SEM image of suspected sensitizer nickel oxide (NiO). Scale bars represent 500 nm in both micrographs. The table below the images lists the quantitative analyses of SiO_2_’s and NiO’s physicochemical properties. The table includes properties as dry powders (e.g., surface area and density as provided by the manufacturer); properties after suspension in ultrapure deionized water (e.g., hydrodynamic diameter and zeta potential and dispersity index), and properties after suspension in cAMEM (cell culture media) (e.g., hydrodynamic diameter, zeta potential, and disperity index. These data were collected using dynamic light scattering.

Figure 2 shows the scanning electron micrographs of SiO_2_ and NiO along with the quantitative physicochemical properties of each material listed in the table. SiO_2_ had an average size of approximately 3 μm, with NiO having an average size of approximately 80 nm. The surface charge of SiO_2_ averaged −56.8 and NiO −9.05, with the hydrodynamic diameter at 1.807 μm for SiO_2_ and 0.963 μm for NiO.

**Figure 3:**
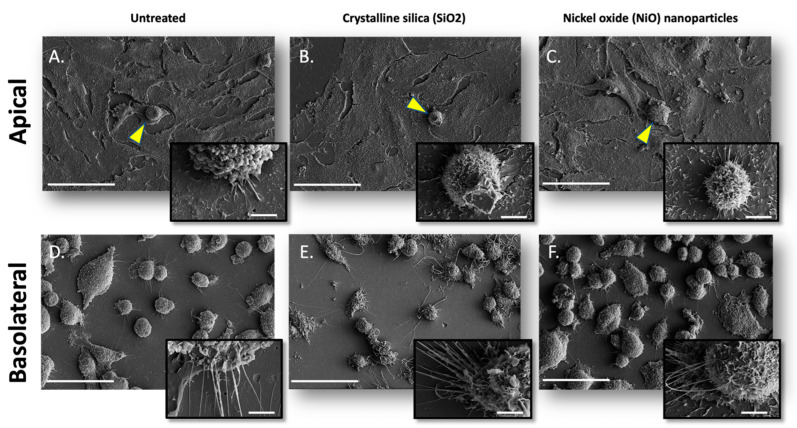
Cell morphology is indicative of cell activation. (Above) Scanning electron micrographs of (**A**) naïve culture, (**B**) SiO_2_-treated culture, and (**C**) NiO-treated culture in an apical chamber. Scanning electron micrographs of (**D**) naïve culture, (**E**) crystalline silica-treated culture, and (**F**) nickel oxide-treated culture in the basolateral chamber. Alveolar macrophages are seen with yellow arrowheads. The scale bar denotes 50 μm. All images were taken at 1200× magnification. Scale bars in large images are 50 µm, while scale bars in inset images are 4 µm.

Figure 3 shows scanning electron micrographs of EC and AM cells in the apical chamber (Figure 3A–C) and DCs in the basolateral chamber (Figure 3D–F). A normal unperturbed epithelial cell structure is seen by confluent monolayers with flattened morphology within untreated cultures (Figure 3A). In contrast, disruption and increased size of epithelial cells within the monolayer (an indication of apoptotic cells) are seen in SiO_2_- and NiO-treated cultures, respectively (Figure 3B,C). Increases in microvillar protrusions on the membrane surface are also visible in SiO_2_- and NiO-treated cultures.

Dendritic cell size and dendrite length increased in the basal compartments (Figure 3D–F) of treated versus untreated cultures. Compared to treated cells, untreated cultures show DCs appearing smaller in size with fewer and shorter dendrites per cell.

**Figure 4:**
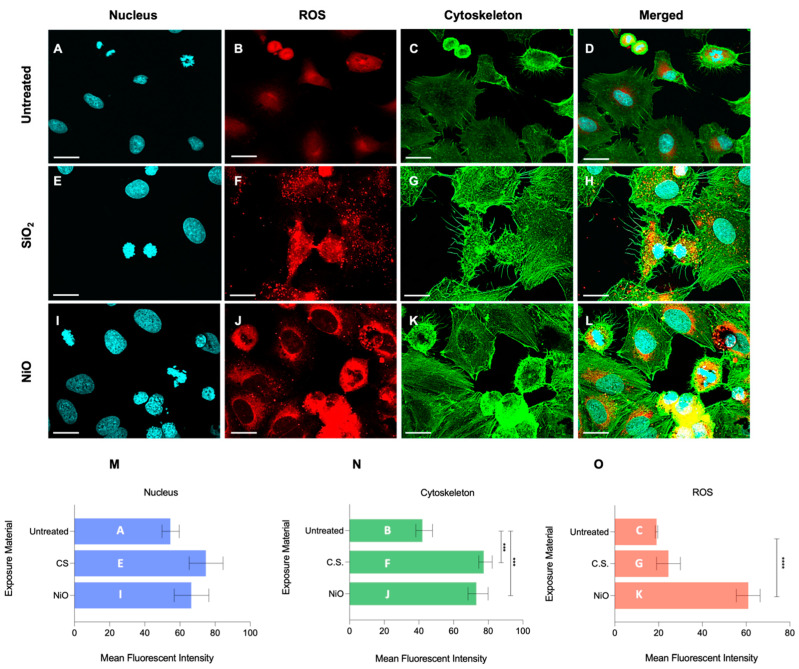
Biochemical analyses via confocal laser scanning micrographs of cells. The nucleus is stained with DAPI (blue), mitochondrial ROS with MitoTracker™ Red CMXRos, and F-actin with ActinGreen™ 488 ReadyProbes™ Reagent. Images were taken at 60× magnification. The scale bar denotes 20 μm. Quantification of fluorescence was performed with CellSens software V4.2. The inset letters of bar graphs (in panels **M**–**O**) correspond to each micrograph label (in panels **A**–**L**). Significance is noted: *** *p* ≤ 0.001, and **** *p* ≤ 0.0001.

Confocal laser scanning microscopy (CLSM) was performed to measure reactive oxygen species (ROS), nuclear binding activity, and cytoskeletal structure. Figure 4 shows micrographs imaging DNA via NucBlue live cell stain, cytoskeleton (F-actin) via ActinGreen 488^®^ ReadyProbes, and mitochondrial ROS via MitoTracker Red CMXRos. Quantification of mean fluorescence intensity (MFI) was assessed using the Olympus CellSens software V4.2. Resultant MFI calculations were compared across untreated (54.71, untreated) vs. SiO_2_-treated (74.87, SiO_2_) vs. NiO-treated (66.61, NiO) cultures. While both treated cultures showed increased nuclear binding activity, no statistical significance was seen. Only NiO treatment induced significant increases in ROS. ROS from all exposures were as follows: untreated, 19.02; SiO_2_-treated, 24.49; NiO-treated, 60.94. F-actin, a measure of proliferation, increased significantly in both SiO_2_- and NiO-treated cultures compared to untreated cultures (untreated, 43.02; SiO_2_, 78.37; NiO, 74.09). NiO treatment showed significant increases in ROS and F-actin compared to untreated, while SiO_2_-treated cultures only showed significant increases in F-actin.

Transcriptome related to innate and adaptive cytokines was performed on delta Ct values and normalized to the reference gene ubiquitin C (UBC) by subtracting gene(s) of interest from the reference gene. Using delta Ct values where higher values represent increased expression, heatmaps were created to compare transcriptomics across the array of cytokine-related genes. A comparison between SiO_2_- and NiO-treated cultures revealed several inflammatory genes associated with inflammatory responses and associated explicitly with cell activation and recruitment (CCL1, CCL3, CNTF, CSF2, FASLG, IL-5, IL-8, OSM, IL-12b, IL-17, LIF, and TNF) were upregulated in NiO treatment relative to untreated and SiO_2_-treated cultures, indicating possible sensitizing potential.

**Figure 5:**
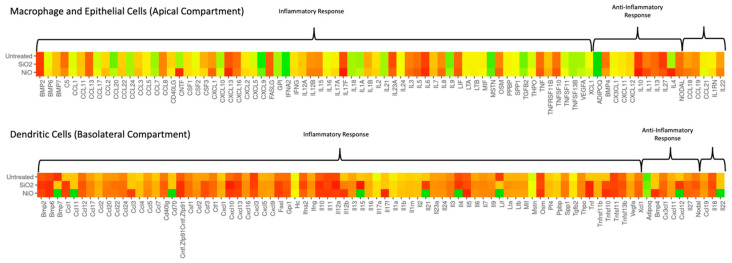
Transcriptome among macrophage and epithelial cells or dendritic cells. Red indicates upregulation and green indicates downregulation. ΔCt values were calculated as follows: ΔCt = Ct*_ref_* − Ct*_goi_*, where *ref* = *reference gene* and *goi* = *gene of interest*. As the ΔCt value decreases, the *goi* expression also decreases.

Figure 5 shows the transcriptome heatmaps for each respective treatment. For ECs and AMs in the apical chamber, the following genes were upregulated in NiO compared to SiO_2_ and untreated cultures: *BMP6*, *CCL1*, *CCL2*, *CCL3*, *CCL17*, *CCL18*, *CCL19*, *CCL20*, *CNTF*, *CXCL1*, *CXCL2*, *CXCL5*, *CXCL9*, *CXCL10*, *CXCL13*, *IL-1RN*, *IL-1α*, *IL-2*, *IL-3*, *IL-4*, *IL-5*, *IL-6*, *IL-7*, *IL-8*, *IL-9*, *IL-10*, *IL-11*, *IL-12β*, *IL-15*, *IL-16*, *IL-17α*, *IL-17F*, *IL-22*, *MSTN*, *OSM*, *TGFβ2*, *THPO*, *TNF*, *TNFRSF11*, *TNFSF10*, *TNFSF11*, *VEGFa*, *ADIPOQ*, *NODAL*.

Downregulated genes from ECs and AMs for NiO compared to SiO_2_ and untreated cultures include: *C5*, *CSF3*, *CD40LG*, *CXCL16*, *CXCL10*, *IFNa2*, *IL-1b*, *IL-23*, *XCL1*, *BMP4*, *IL-27*, and *CCL21*. DCs in the basolateral chamber showed that the following genes were upregulated in NiO-treated cultures compared to SiO_2_-treated and untreated cultures: *CCL3*, *CCL20*, *CCL24*, *CSF2*, *IL-5*, *IL-11*, *IL-12b*, *IL-17F*, *OSM*, TNFSF10, *TNFSF11*, *BMP4*, and *CX3CL1*. Genes downregulated in DCs in NiO-treated cultures include *ADIPOQ*, *BMP7*, *CD70*, CXCL3, *IL-4*, *IL-15*, *IL-21*, *IL-22*, *LIF*, and *CXCL12*.

To better understand which biological pathways may be perturbed, genes were subsequently loaded to the david.ncifcrf.gov database, and KEGG pathways were investigated to examine potential biological consequences. Table 1 and Table 2 show specified pathways from KEGG analyses, which genes were up- or downregulated, and the possible biological outcomes from the perturbed genes within the pathway analyzed. Genes from AMs and ECs following NiO treatment compared to untreated cultures corresponded to pathways associated with chemokine signaling, cytosolic DNA sensing, rheumatoid arthritis, Toll-like receptor signaling, Jak-STAT signaling, inflammatory bowel disease, RIG-I-like receptor signaling, type I diabetes mellitus, asthma, PI3K-Akt signaling, T cell receptor signaling, NF-κB signaling, TGF-β signaling, NOD-like receptor signaling, natural killer cell-mediated cytotoxicity, and TNF signaling (Table 1).

Genes in DCs following NiO treatment corresponded to the following pathways: chemokine signaling, cytosolic DNA-sensing, rheumatoid arthritis, Toll-like receptor signaling, Jak-STAT signaling, inflammatory bowel disease, RIG-I-like receptor signaling, type I diabetes mellitus, asthma, PI3K-Akt signaling, and T cell receptor signaling (Table 2).

**Figure 6:**
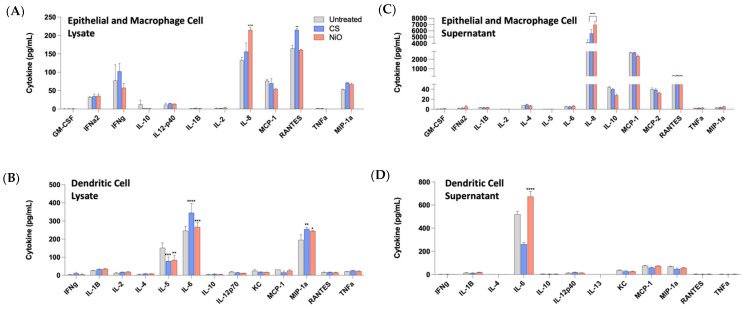
Luminex data for epithelial and macrophage cells (**A**,**B**) and dendritic cells (**C**,**D**). All cytokines are reported in pg/mL. Significance is to untreated samples and is denoted with an * (*, *p* ≤ 0.05; **, *p* ≤ 0.01; ***, *p* ≤ 0.001; ****, *p* ≤ 0.0001).

Luminex was performed on both culture supernatant and cell lysate at 24 h post-exposure to measure an array of cytokines associated with inflammation. The results indicate increases in protein expression common to inflammation and related to cell infiltration, activation, and maturation (Figure 6). The cell supernatant and lysate of ECs and AMs showed significant increases in IL-8 for NiO-treated cultures. At the same time, SiO_2_ also showed significant increases in RANTES from the cell lysate compared to NiO and untreated cultures. The cell lysates of DCs showed significant increases in IL-6 and MIP-1a in NiO- and SiO_2_-treated cultures compared to untreated cultures. Significant decreases were seen in IL-5 from NiO- and SiO_2_-treated cultures compared to untreated cultures. Supernatants from DCs showed only a significant increase in IL-8 for NiO compared to both SiO_2_ and untreated cultures, indicating a prolonged recruitment of neutrophils (Figure 5).

**Figure 7:**
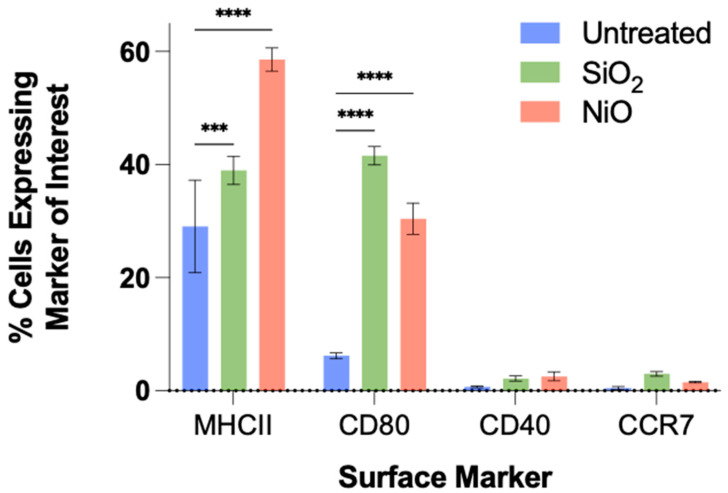
Dendritic cell activation markers. Flow cytometry data for surface markers of MHCII, CD80, CD40, and CCR7 were measured. All samples were analyzed in the live population only. Significance is to untreated cultures, denoted with *** *p* ≤ 0.001; **** *p* ≤ 0.0001.

Flow cytometry was performed to identify specific DC markers related to activation and antigen presentation (CD40, MHCII, CD80) and migration (CCR7) (Figure 7). MHCII expression was significantly upregulated for both SiO_2_- and NiO-treated cultures compared to untreated cultures (29.07% for untreated; 38.97% for SiO_2_-treated; 58.57% for NiO-treated). CD40 expression was increased in both SiO_2_- (2.16%) and NiO-treated cultures (2.53%) compared to untreated cultures (0.66%), but not significantly so. CD80 expression was significantly increased in both SiO_2_- and NiO-treated cultures compared to untreated cultures (41.57%, 30.4%, and 6.18%, respectively). CCR7 expression was increased in both SiO_2_- (2.98%) and NiO-treated cultures (1.51%) when compared to untreated cultures (0.46%), but the results did not reach statistical significance.

## 3. Discussion

The local milieu of the lungs is designed to be anti-inflammatory to prevent excessive inflammation and exacerbated immune responses to every exogenous material inhaled. Specifically, alveolar macrophages (AMs) phagocytose and continually patrol the lumen of the alveolar spaces where they engulf and dispose of foreign materials. In the steady state, AMs are suppressive by secreting immunosuppressive cytokines to surrounding cells [33,34,35]. Dendritic cells (DCs) are the primary antigen-presenting cells throughout the human system. When activation occurs, they can extend their dendrites through the tight junctions of the epithelial barrier and into the luminal space, where they recognize, capture, and process antigens [36,37]. Once activated, DCs will upregulate co-stimulatory markers and migratory receptors, which are necessary for traveling to local lymph nodes and eliciting an activating and sustained response from T and B cells to form a lasting immune response [38]. The formation of antigen-specific T and B cells can ultimately lead to sensitization to any xenobiotic. Therefore, cell activation and maturation mechanisms can potentially lead to detecting early biomarkers of respiratory sensitizing potential.

While there are no current biosignatures of respiratory sensitization common to all known respiratory sensitizers, there are general principles of sensitization that appear to hold for most known sensitizers at the respiratory junction: neutrophil influx and general cell activation of recruited cells [39]. The cytokine milieu within the lungs determines the effector function of immune cells, specifically regarding allergy and sensitization. Because neutrophils are commonly recruited as a first-line defense against various cell and tissue assaults, their use as biomarkers is currently limited without additional endpoints simultaneously measured. This study examined the effects of particulates on cells, and the data obtained can assist in identifying biosignatures linked to respiratory sensitization. The observations made can be useful for future studies with differing experimental approaches.

During respiratory sensitizing reactions to chemical sensitization, our previous study found that specific cytokine-related genes, including CXCL5, IL-6, IL-8, and CCL7, were expressed in a perturbed manner [6]. The CXC chemokine ligand 5 (CXCL5) is known to be a potent neutrophil attractant both in vivo and in vitro and is known to be secreted by both innate (e.g., ECs) and adaptive (CD4 T cells) immune cells [40,41,42]. Several known pathologies are associated with increased expression of CXCL5, including COPD from cigarette smoking, infections, and allergy [41,42,43,44,45,46,47]. Interleukin 6 (IL-6), a pleiotropic cytokine capable of both inflammatory and anti-inflammatory responses, can elicit chronic inflammation and allergy in the lungs [48,49]. While various cell types can secrete IL-6 at the onset of insult or injury, it has recently been revealed that pulmonary DCs and AMs are specific cytokine sources for inflammatory conditions such as sensitization and allergic airway inflammation [50]. Another potent neutrophil attractant, interleukin 8 (IL-8), is secreted early during the inflammatory process by both ECs and AMs [51,52,53]. Importantly, IL-8 has been shown to increase various respiratory diseases in both in vivo and in vitro studies [39,54,55,56]. C-C chemokine ligand 7 (CCL7) is a powerful attractant for eosinophils and affects neutrophils and epithelial cells. Its expression increases in respiratory allergy, airway hyperresponsiveness, and sensitization. Furthermore, exposure to particulates, especially suspected respiratory-sensitizing particulates, leads to increased levels of the inflammatory protein chemokine ligand 3 (CCL3) [57,58]. This protein is secreted by different cell types, such as ECs, AMs, and DCs, and has been observed to release cytokines previously seen with chemical sensitizers [59]. Mast cells and eosinophils are activated by CCL3, which is a potent trigger. These cells contribute significantly to lung inflammation in conditions such as allergies and airway hyperresponsiveness [59,60,61]. Studies conducted in living organisms have demonstrated that exposure to NiO nanoparticles can lead to an increase in neutrophil and eosinophil counts [23,24]. Although the current study did not measure cellular influx, it did evaluate the rise in transcripts and cytokines related to cellular influx and activation. The results of this study are consistent with those observed in in vivo studies. Similarly, in vivo studies examining SiO2 have revealed an increase in inflammatory cytokines, such as IL-6, with little to no change in total IFN-gamma, which is in line with the findings of the current study [62]. Overall, the data suggest that the alveolar model used is comparable to animal models using similar exposure materials.

Unlike skin sensitization, the lungs lack a validated model that accurately identifies known or potential sensitizers [63]. The current gold standard uses animal models where the local lymph node assay (LLNA) and serum cytokine levels are the primary methods for assessing sensitization. Still, the LLNA is the only universally approved technique for dermal testing. It is important to note that the cytokine levels are unreliable as different animal models have different immune systems and subsequent responses and poor translation to human immune responses [8,63,64,65,66,67]. Recent investigations into respiratory sensitization have attempted to use the skin sensitization assays of the direct peptide reactivity assay (DPRA) and the peroxidase peptide reactivity assay (PPRA). However, while these methods show promise, they need to be more accurate on their own (accuracy ~80%) for the utilization [68,69]. As such, it is necessary to develop methods for identifying and assessing the respiratory sensitizing potential of both current and novel materials.

As an alternative to animal testing and to circumvent many of the issues associated with failure to translate to humans, human-derived cells can and should be a current method of investigation [1,14,15]. Studies have used cells that closely mimic DCs or single-cell types (DCs) rather than multi-cell models capable of introducing intercellular communication and responses [38]. While promising results have been shown, a lack of high sensitivity, specificity, and accuracy in predicting outcomes precludes the use of single-cell systems for now. Including multiple cell types and various techniques designed to probe multiple endpoints may improve the specificity, sensitivity, and accuracy of any lung models in development.

This study showed an alveolar cell culture model mimicking in vivo architecture to differentiate responses induced by a known respiratory irritant (SiO_2_) and a suspected respiratory sensitizer (NiO). Endpoint measurements included: (1) Cell morphology measured by microscopy; (2) Transcriptomics measured by real-time polymerase chain reactions (rt-PCR); (3) Cytokine profiling and expression measured by a Luminex multiplex assay; (4) Expression of cell surface markers measured via flow cytometry; and (5) Biological pathway analyses probed via the Database for Annotation Visualization and Integrated Discovery (DAVID). Respiratory sensitization typically requires an initial exposure, induction, subsequent re-exposure, and elicitation, for typical symptomatic responses. However, specific biochemical (surface marker, cytokine, and gene) responses are required for innate cells to recruit and activate immune-specific adaptive cells (e.g., T and B cells). Because of this requirement, it is hypothetically possible to identify respiratory sensitizers before the elicitation phase by examining innate cells at the exposure site. This would allow for the development of a rapid assay capable of predicting sensitizing potential before exposure, preventing the implementation of novel materials that may lead to poor health outcomes. Some cytokines, such as IL-8, peak 24 h post-exposure [70]. Additionally, it has been shown that activation markers of DCs increase in expression as a function of time after exposure [71]. To best account for changes in cell marker expression, transcriptome, and cytokine release associated with known sensitization potential (i.e., cell recruitment, initiation, and activation), a timepoint measurement of 24 h post-exposure was chosen.

Dendritic cells (DCs), the primary antigen-presenting cells, are critical to immune responses throughout the body and are essential to eliciting long-term immune responses. On activation, these cells will readily take up and process exogenous material, increase the surface expression of MHCII, where the foreign antigen is presented, and migrate to local draining lymph nodes to train and activate T and B cells [72,73,74,75,76]. Furthermore, DCs will increase the biosynthesis of costimulatory molecules (CD40 and CD80), which bind to T cells for effector phenotyping in lymph nodes [77,78]. Results from this study show significant increases in MHCII and CD80, as well as trends towards increased expression levels of CD40 and the migratory receptor CCR7 after exposure to a suspected respiratory sensitizer (NiO).

In ECs and AMs, perturbations in the transcriptome are related to biological pathways, which can affect immune cell recruitment, proliferation, differentiation, and survival; increases in ROS production and cytoskeletal component changes; cellular migration, and activation of fibroblasts. Biologically, these pathways affect acute and chronic inflammatory responses, the ability of lymphoid cells to home relevant tissues of interest, and cell signaling. Downregulated transcriptomic profiles in ECs and AMs lead to perturbed biological pathways, which can cause decreases in cell cycling and cell-effector functionality.

In the lungs, it has been shown that excessive increases in oxidative stress can hinder AM functionality, leading to pathologic inflammation [79]. While many regulatory mechanisms prevent undue oxidative stress, one of the most common methods to assess recovery or continued insult is to measure glutathione levels. Glutathione (GSH) concentrations are relatively high in the extracellular fluid within the lung compartment, purportedly to reduce oxidative stress [80]. GSH levels tend to peak at 24 h post-exposure [81]. Results showed significant increases in ROS after exposure to the suspected sensitizer NiO at 24 h post-exposure, indicating that not only had cellular mechanisms not compensated for the injury, but that an increased likelihood for severe pathologic inflammation exists.

In dendritic cells, it has been shown that a known sensitizer will cause upregulation of the major histocompatibility complex (MHC) class II, co-stimulatory molecules (e.g., CD40, CD80, and CCR7), and inflammatory cytokines [82]. Results from this study showed several morphological changes after exposure to either a known irritant or suspected respiratory sensitizer (SiO_2_ and NiO, respectively). Further changes were seen when comparing the suspected sensitizer NiO to the irritant SiO_2_ and untreated cultures. Significant increases in cytokine production, perturbations in the transcriptome, and surface marker expression related to inflammation, allergy, and sensitization were all noted after exposure to NiO. Taken together with previous studies, this study helps further the idea that various endpoint readouts (morphology, transcriptomics, cytokine production, and cell surface markers) can help establish a high throughput assay capable of assessing the sensitizing potential of new and existing substances.

### Limitations and Suggestions for Model Design

The lungs are highly complex and constantly changing. Current models do not account for important processes such as cell turnover, activation, and communication between cells. Additionally, there are barriers such as fluids, such as surfactant and mucus, that capture and eradicate foreign substances but are not present in current models. To improve accuracy, studies should use fluidic devices and cell migration assays and include T and B cells to assess the activation of immune cells. While in vitro models have limitations compared to live organs or organisms, they offer the ability to study specific mechanisms and test new materials and contaminants. This can lead to significant advancements in understanding and treating lung-related issues.

## 4. Materials and Methods

*Experimental premise.* To better understand the utility of this model, a model comparing a suspected respiratory sensitizing particulate (e.g., nickel oxide, NiO) and a known respiratory irritant (e.g., crystalline silica, SiO_2_) were utilized. SiO_2_ and NiO were made in-house with physicochemical properties shown in Figure 2.

*Reagents.* A commercially available engineered nickel oxide particle was purchased from Nanoshel, LLC (County Cavan, Ireland; Product No. NS6130-03-337). Similarly, a commercially available engineered silicon dioxide particle was purchased from Sigma-Aldrich (St. Louis, MO, USA; Product No. S5631).

*Physicochemical characterization of the materials.* Hydrodynamic diameter, polydispersity, and zeta potential measurements were taken using a Zetasizer Nano ZS (Malvern Instruments Ltd., Westborough, MA, USA). All measurements were performed in triplicate with sample parameters for absorbance and refractive indices set to 0.01 nm and 1.580, respectively.

*Nanoparticle preparation for cell culture studies.* Samples were diluted to 0.002 wt% in triplicate. Dilutions were performed in phenol-free cell culture media.

*Cell culture.* A549 epithelial cells and U937 monocytes were grown in complete RPMI (cRPMI) 1640 (Thermo Fisher Scientific Inc., Waltham, MA, USA) and supplemented with 10% FBS and 1% penicillin-streptomycin. JAWSII cells were cultured in complete Alpha minimum essential medium (cAMEM) with nucleosides (ThermoFisher Scientific Inc., Waltham, MA, USA) and supplemented with 5 ng/mL murine GM-CSF (BioLegend, San Diego, CA, USA); 20% fetal bovine serum, and 1% penicillin-streptomycin. All cells were maintained at 37 °C in a humidified 5% CO_2_ atmosphere until ready for use.

Cells were plated as previously described [83]. A549 epithelial cells were added to 12-well plates fitted with polyethylene terephthalate (PET) Transwell^®^ membranes (Corning, Tewksbury, MA, USA) at 28 × 10^4^ cells/cm^2^. Cells were allowed to adhere for 2–3 days until a confluent monolayer was formed. Media were removed, and inserts were inverted and placed into sterile glass dishes. JAWSII cells were resuspended in 500 μL of cAMEM and plated on the basal surface of the membrane at 7 × 10^4^ cells/cm^2^ and allowed to adhere for hours. After excess media were removed, inserts were reverted into the well plate, and 1 mL of cAMEM was added to the basolateral chamber. U937 macrophages were added at a 1:9 ratio of U937:A549 in cRPMI, and the apical chamber was replenished to 500 μL [84]. The model was then placed in a 37 °C humidified incubator at a 5% CO_2_ atmosphere to allow cells to rest for 24 h before exposure.

*Macrophage differentiation.* U937 monocytic cells were incubated with 100 ng/mL phorbol 12-myristate-13-acetate (PMA) for 24 to 48 h, as previously described [85]. The cells were washed two times in sterile 1X PBS, and fresh media were added. Cells rested in the 37 °C humidified incubator at a 5% CO_2_ atmosphere for 72 h before use. The adherent cells were dissociated using trypsin, resuspended in cRPMI, counted, and plated according to use.

*Chemical exposure.* All exposure materials were added to the apical chamber of the Transwell^®^ membrane. SiO_2_ was added at 50 ppm and was a positive control for cellular irritation. NiO was added at 50 ppm as a test compound for suspected sensitization. The post-exposure period was 24 h to assess early markers of respiratory sensitizing potential.

*Cell imaging.* Imaging occurred as previously described [6]. Briefly, Transwell^®^ plates were removed from the incubator, and cells from both chambers were washed twice with 1X phosphate-buffered saline (PBS) solution. Glutaraldehyde, at a concentration of 1:10 in 1X PBS, was added to both chambers for 10 min and followed by three washes of 1X PBS at 10 min intervals. PBS was replaced with 4% osmium tetroxide in PBS for 2.5 h at 4 °C. Three consecutive wash steps were repeated, followed by a series of dehydration steps that occurred twice, each at 10 min intervals: 50% ethanol (EtOH); 70% EtOH; 90% EtOH; 100% EtOH. The well inserts were removed and submerged in 100% EtOH in sequence. The membranes were carefully excised with a razor blade, placed into sterile buckets, and dried in a critical point dryer (CPD300, Leica, Buffalo Grove, IL, USA). Imaging was performed on a focused ion beam scanning electron microscope (FIB-SEM, Versa 3D, FEI ThermoFisher Scientific, Hillsboro, OR, USA) at 5 kV with a spot size of 5.0 and a working distance of 10 mm using an Everhart–Thornley *detector*.

Cells were stained with NucBlue^TM^ live cell stain (ReadyProbes, Thermo Fisher Scientific Inc.), MitoTracker™ Red CM-H_2_Xros (Thermo Fisher Scientific Inc.), and ActinGreen^TM^ 488 ReadyProbes reagent (Thermo Fisher Scientific Inc.) for analysis of the nucleus, reactive oxygen species (ROS), and F-actin cytoskeleton, respectively. Images were captured using a confocal laser scanning microscope (FV-3000, Olympus Corp., Center Valley, PA, USA). Quantifying fluorescence was performed with CellSens software V4.2 on a Wacom Cintiq 22HD workstation (Olympus Corp.).

*Transcriptomics*. Polymerase chain reaction (PCR) plates for a panel of innate and adaptive cytokine were purchased (AB Applied Biosystems TaqMan^®^ Array 96-well plates) for mouse and human (catalog # 4391524). The Transwell^®^ compartments were evaluated separately by collecting cells and supernatant from both the apical and basal sides. Macrophage and epithelial cells were analyzed from the former, while the latter was used for dendritic cells. RNA collection, cDNA formation, and plating protocols followed manufacturer instructions. Plates were assessed on a QuantStudio 6 Flex RealTime PCR system (ThermoFisher Scientific), delta Ct values were calculated (i.e., ∆Ct = Ct (gene of interest)—Ct (housekeeping gene)), and heatmaps were created. Only ∆Ct values greater than 0.5 were considered for statistical analyses.

Analysis of PCR data was performed using the Database for Annotation, Visualization, and Integrated Discovery (DAVID v6.8) [86]. Briefly, gene lists from each assay were sorted based on the official gene symbols. Once sorted, pathways were identified utilizing the Kyoto Encyclopedia of Genes and Genomes (KEGG, Kenoisha laboratories, Tokyo, Japan) (https://www.genome.jp/kegg/) pathway analyses.

*Cytokine and chemokine multiplex analysis.* Using the manufacturer’s instruction, cytokines and chemokines were measured using Milliplex MAP cytokine/chemokine magnetic bead panels for both mouse and human (Millipore, Burlington, MA, USA). Fluorescence was analyzed using the Bio-Plex Luminex 100 XYP (Bio-Rad, Hercules, CA, USA) with the Bio-Plex Manager 4.1 software. Subsequently, a 5-parameter curve-fitting algorithm was applied for standard curve calculations.

*Flow cytometry.* Fluorochrome-conjugated antibodies to CD40 (3/23), I-A/I-E (M5/114.15.2), CCR7 (4B12), and CD80 (B7-1) were purchased from BioLegend (San Diego, California, USA). TruStain FcX™ (anti-mouse CD16/32) was used to block against non-specific Fc binding, and 7-amino-actinomycin D (7-AAD) was used to measure live/dead cells. A live/dead cell gating was obtained, and the analysis was performed on a FACSVerse (BD Biosciences, Franklin Lakes, NJ, USA) with a subsequent analysis performed utilizing FlowJo v10. Cells were prepared as follows: after 24 h, the media were removed from the basal chamber, and 0.25% trypsin-EDTA (Fisher Scientific) was added for 5 min in a 37 °C humidified incubator at a 5% CO_2_ atmosphere. Equal parts of complete media were added to each well and mixed to resuspend cells. The samples were then spun in a temperature-controlled incubator at 4 °C, and the supernatant was removed. After removing the supernatant, cells were washed and spun before cell staining. Staining was performed as previously described [87]. Briefly, cells were washed with FACS buffer (2% FBS, 0.1% NaN_3_ in PBS), blocked with anti-mouse CD16/32 (93), and placed on ice for 10 min. Consecutively, the cells were stained on ice for 45 min using anti-CD40, I-A/I-E, CD80, and CCR7 at 1:200 dilutions in FACS buffer. Lastly, the cells were washed three times in a FACS buffer and resuspended to a final volume of 0.2 mL before a FACS analysis.

*Dynamic light scattering (DLS) analysis.* Particles were suspended in phenol-free cell culture media. Hydrodynamic diameter, dispersity, and zeta potential measurements were taken using a Zetasizer Nano ZS (Malvern Instruments Ltd., Westborough, MA, USA). All measurements were performed in triplicate with sample parameters for absorbance and refractive index set to 0.01 nm and 1.580, respectively.

*Statistical Analyses*. Unless otherwise noted, all samples were performed in triplicate with three replicates for each methodology for nine samples in each experimental setup. Data were analyzed using analysis of variance (ANOVA) followed by a *t*-test using Microsoft Excel v16.72 and GraphPad Prism 9.4.1. Significance is noted in the figure caption where applicable, with data presented as mean with ± standard deviation.

## 5. Conclusions

While it is possible that any chemical or particulate can lead to sensitization within the respiratory system leading to lifelong allergies, hypersensitivity, and other complications, the incidence rate is still low within the more significant population. However, to prevent poor health outcomes, especially in areas of lower development, assessing for respiratory sensitization is a continued focus in immunotoxicology. To provide preventative, protective, or curative responses, it is critical to understand the processes that promote long-term immune reactions before, during, and/or after toxicant exposure.

The model utilized in this study can rapidly adjust cell types to mimic the area of the lung (i.e., upper or lower lung) to be studied. The simplicity of design, low cost of setup, ability to switch to an air–liquid interface if needed, and the ability to modify the endpoints measured are all strengths in using the model herein. While this model is static and does not include adaptive immune cells, subsequent studies are needed. They are underway to assess the ability of the cells in this model to activate and recruit T and B cells after exposure to known respiratory sensitizers and novel materials.

Although submerged conditions are still commonly used in most studies, air–liquid interface (ALI) cultures have proven increasingly successful in recent years [88]. However, many labs are still unable to use ALI due to the high cost and limited availability of the necessary equipment and aerosol technology. It is crucial for labs to have easy access to the equipment and aerosol technology needed to make a universal assay capable of assessing respiratory sensitizing potential. Submerged systems are the optimal choice until ALI technology and equipment become more readily available. If a submerged system can differentiate between known respirable sensitizers and non-sensitizers, it is preferred due to its ease of use. This study used a submerged system and two particulates to achieve this task by successfully differentiating a known non-sensitizing particulate from a suspected sensitizer with supporting evidence in the clinical literature.

When selecting and optimizing a co-culture system, the type of cell used is crucial. Although some human dendritic cell lines are available, they are not well-established in the literature and can be difficult to obtain. Obtaining human PBMCs that have differentiated into dendritic cells is also challenging, expensive, and can vary significantly between individuals. Therefore, researchers often use cell lines to ensure response consistency and simplify the validation process. Studies have demonstrated that murine dendritic cells exhibit similar responses to human dendritic cells, with JAWSII cells being a good example [1,89,90]. Instead of using human monocytic cell lines, immature dendritic cell lines that do not require differentiation or marker validation can streamline the assay development process and make it easier to validate results across multiple laboratories.

## Figures and Tables

**Table 1:** DAVID pathway analysis for epithelial and macrophage cells in the apical compartment. The table includes the pathways of up- and down-regulated genes and possible biological consequences of regulation. Only genes with ΔCt values > 0.5 for comparisons of naïve vs. SiO_2_ vs. NiO treatments were considered for analysis.

Specified Pathway	Regulation	Cytokines	Biological Consequence Related to Sensitization
Chemokine Signaling	UP	CCL1, CCL2, CCL3, CCL5, CCL7, CCL17, CCL18, CCL19, CCL20, CCL22, CXCL1, CXCL2, CXCL5, CXCL9, CXCL10, CXCL13, PPBP	Cell infiltration, growth, survival, differentiation, ROS production, cytoskeletal changes, leukocyte migration
	DOWN	CCL8, CCL11, CCL13, CCL21, CCL24, CXCL11, CXCL12, CXCL16, CX3CL1, XCL1,	Inhibition of cell cycling
Cytosolic DNA-sensing pathway	UP	CXCL5, CXCL10, IL-1b, IL-6, IL-18	Production of pro-inflammatory cytokines, type I interferons, NK cell activation
	DOWN	IFNa2	Decreased NK cell activation, improved cell survival
Rheumatoid arthritis	UP	CCL2, CCL20, CCL3, CCL5, CXCL1, CXCL2, CXCL5, CSF1/2, IFNg, IL-1a, IL-1b, IL-6, IL-11, IL-15, IL-17a, IL-18, LTB, TGFb2, TNF, VEGFa, TNFSF11, TNFSF13b	Fibroblast activation, angiogenesis, VEGFa signaling, leukocyte migration, inflammatory cell infiltration
	DOWN	CXCL12, IL-23a	Decreased inflammatory cell responses, decreased vasculature permeability
Toll-like receptor signaling pathway	UP	CCL3, CCL5, CXCL9, CXCL10, IL-1b, IL-6, IL-12a, IL-12b, SPP1, TNF	Production of inflammatory cytokines, T cell stimulation and recruitment
	DOWN	INFa2, CXCL11	decreased TH2 response
Jak-STAT signaling pathway	UP	LIF, CNTF, CSF2, IFNg, IL-2, IL-3, IL-4, IL-5, IL-6, IL-7, IL-9, IL-10, IL-11, IL-12a, IL-12b, IL-15, IL-21, IL-22, IL-24, OSM, THPO	Cell proliferation, differentiation, survival
	DOWN	IFNa2, CSF3, IL-13, IL-23a, IL-27	Decreased cell cycling
Inflammatory bowel disease	UP	IFNg, IL-1a, IL-1b, IL-2, IL-4, IL-5, IL-6, IL-10, IL-12a, IL-12b, IL-17a, IL-17f, IL-18, IL-21, IL-22, TGFb2, TNF	Inflammatory pathways and autoimmune responses, T helper (Th) 1, 2, 17 differentiation
	DOWN	IL-13, IL-23a	Decrease in T helper (TH) 1 and 17 effector cells, regulatory T cells, and NKT cells
RIG-I-like receptor signaling pathway	UP	CXCL10, IFNa2, IL-12a, IL-12b, TNF	Inflammatory cytokines, type 1 interferons, protein synthesis, dendritic cell activation, NK cell activation, cytotoxic T lymphocyte (CTL) differentiation, antibody production
Type 1 diabetes mellitus	UP	FASLG, IFNg, IL-1a, IL-b, IL-2, IL-12a, IL-12b, LTA, TNF	Upregulation of MHCII, macrophage activation, cytotoxic T lymphocyte (CTL) differentiation, CD4 T cell activation
Asthma	UP	CD40lg, IL-3, IL-4, IL-5, IL-9, IL-10, TNF	Lung epithelial cell and fibroblast activation, T helper cell 2 differentiation and B cell interactions, mast cell activation, eosinophil recruitment and activation
	DOWN	CCL11, IL-13	Decrease in smooth muscle cell recruitment and repair, decrease in eosinophil recruitment
PI3-Akt signaling pathway	UP	FASLG, CSF1, IFNa2, IL-2, IL-3, IL-4, IL-6, IL-7, OSM, SPP1, VEGFa	Cell proliferation, DNA repair, angiogenesis, cell survival
	DOWN	CSF3	Decreases in cell survival
T cell receptor signaling pathway	UP	CD40lg, CSF2, IFNg, IL-2, IL-4, IL-5, IL-10, TNF	Proliferation, differentiation, immune response, PI3-Akt and Nf-kappa B pathway activation
NF-kappa B signaling pathway	UP	CCL19, CXCL1, CXCL2, CD40lg, TNFSF11, TNFSF13b, IL-1b, LTA, LTB, TNF	Auto-ubiquitination, cell survival
	DOWN	CCL13, CCL21, CXCL12	Decreased CD8 T-cell homing, decreased epithelial cell repair after lung injury
TGF-beta signaling pathway	UP	BMP2, BMP6, BMP7, IFNg, TGFb2, TNF, NODAL	Iron metabolism, transcription factor activation, ubiquitin-mediated proteolysis
		BMP4	Decreased T cell differentiation, decreased iron metabolism
NOD-like receptor signaling pathway	UP	CCL2, CCL5, CXCL1, CXCL2, IFNa2, IL-1b, IL-6, IL-18, TNF	Proinflammatory cytokine release, NLRP3 inflammasome activation
Natural killer cell mediated cytotoxicity	UP	FASLG, TNFSF10, CSF2, IFNa2, IFNg, TNF	Inflammatory cytokine release, release of granules from granulocytes
TNF signaling	UP	CCL2, CCL5, CCL20, CXCL1, CXCL2, CXCL5, CXCL10, LIF, CSF1/2, IL-1b, IL-6, IL-15, LTA, TNF	Leukocyte recruitment and activation, inflammatory cytokine release, cell survival
	DOWN	CX3CL1	Decreased leukocyte recruitment and activation

**Table 2:** DAVID pathway analysis for dendritic cells in the basolateral compartment. The table includes the pathways of up- and down-regulated genes and possible biological consequences of regulation. Only genes with ΔCt values > 0.5 for comparisons of naïve vs. SiO_2_ vs. NiO treatments were analyzed.

Specified Pathway	Regulation	Cytokines	Biological Consequence Related to Sensitization
Chemokine signaling	UP	XCL1, CCL1, CCL12, CCL17, CCL19, CCL2, CCL20, CCL22, CCL24, CCL3, CCl4, CCL5, CCL7, CXCL1, CXCL10, CXCL11, CXCL13, CXCL16, CXCL5, CXCL9, CX3CL1, PF4, PPBP	Cell infiltration, growth, survival, differentiation, ROS production, cytoskeletal changes, leukocyte migration
	DOWN	CCL11, CXCL12, CXCL3	Inhibition of activated granulocytes
Cytosolic DNA-sensing pathway	UP	CCL4, CCL5, CXCL10, IFNa2, IL-1b, IL-18, IL-6	Production of pro-inflammatory cytokines, type I interferons, NK cell activation
Rheumatoid arthritis	UP	CCL12, CCL2, CCL20, CCL3, CCL5, CXCL1, CXCL5, CSF1/2, IFNg, IL-1a, IL-1b, IL-11, IL-17a, IL-18, IL-23a, IL-6, LTB, TGFB2, TNFSF11, TNFSF13b, TNF, VEGFa	Fibroblast activation, angiogenesis, VEGFa signaling, leukocyte migration, inflammatory cell infiltration
	DOWN	CXCL12, CXCL3, IL-15,	Decreases in autocrine function of self-reactive Th1 cells, decreases in Th17 differentiation, decreases in blood vessel permeability
Toll-like receptor signaling pathway	UP	CCL3, CCL4, CCL5, CXCL10, CXCL11, CXCL9, IFNa2, IL-1b, IL-12a, IL-12b, IL-6, SPP1, TNF	Chemotaxis of leukocytes, T cell stimulation and recruitment
Jak-STAT signaling pathway	UP	CTF1, CNTF, CSF2/3, IFNa2, IFNg, IL-10, IL-11, IL-12a, IL-12b, IL-13, IL-2, IL-23a, IL-24, IL-27, IL-3, IL-5, IL-6, IL7, IL-9, OSM, THPO	Cell proliferation, differentiation, survival
	DOWN	IL-15, IL-21, IL-22, IL-4, LIF	Decreases in cell cycling, proliferation, differentiation, and survival
Inflammatory bowel disease	UP	IFNg, IL-1a, IL-1b, IL-10, IL-12a, IL-12b, IL-13, IL-17a, IL-17f, IL-18, IL-2, IL-23a, IL-5, IL-6, TGFB2, TNF	Inflammatory pathways and autoimmune responses
	DOWN	IL-21, IL-22, IL-4,	Decrease in T helper (TH) 1 and 17 effector cells, regulatory T cells, and NKT cells
RIG-I-like receptor signaling pathway	UP	CXCL10, IFNa2, IL-12a, IL-12b, TNF	Protein synthesis, dendritic cell activation, NK cell activation, cytotoxic T lymphocyte (CTL) differentiation, antibody production
Type 1 diabetes mellitus	DOWN	FASL, IFNg, IL-1a, IL-1b, IL-12a, IL-12b, IL-2, LTA, TNF	Decreases in cytotoxic CD8+ T cells
Asthma	UP	CD40lg, IL-10, IL-13, IL-3, IL-4, IL-5, IL-9, TNF	Decreases in mast cell activation
	DOWN	CCL11	Decrease in smooth muscle cell recruitment and repair, decrease in eosinophil recruitment
PI3-Akt signaling pathway	UP	FASL, CSF1, CSF3, IFNa2, IL-2, IL-3, IL-6, IL-7, OSM, SPP1, VEGFa	Cell proliferation, DNA repair, angiogenesis, cell survival
	Down	IL-4	Decreases in cell survival
T cell receptor signaling pathway	UP	CD40LG, CSF2, IFNg, IL-10, IL-2, IL-4, IL-5, TNF	Proliferation, differentiation, immune response, PI3-Akt and Nf-kappa B pathway activation

## Data Availability

Data are available upon request.
